# Non-Contact Acoustic Emission Monitoring of Corrosion under Marine Growth

**DOI:** 10.3390/s23010161

**Published:** 2022-12-23

**Authors:** Sarjoon Alkhateeb, Filippo Riccioli, Felipe Leon Morales, Lotfollah Pahlavan

**Affiliations:** 1 Department of Maritime and Transport Technology, Delft University of Technology, 2628 CD Delft, The Netherlands; 2 CORROSION Laboratory, 2751 DB Moerkapelle, The Netherlands

**Keywords:** acoustic emission, corrosion, damage detection, localisation, marine growth

## Abstract

Offshore support structures and mooring systems are predominantly subject to corrosion and fatigue. These structures are typically covered with marine growth of various types. Conventional inspection methods for assessment of the structural integrity require access to the cleaned surface of these structures; however, the cleaning process is highly undesirable from the technical, economical, and environmental points of view. This paper highlights research on feasibility assessment of detection and localization of corrosion damage under marine growth using acoustic emission (AE). Experiments were conducted on two carbon steel plates, one baseline sample and one covered with artificially fabricated marine growth. The results of accelerated corrosion experiments suggest that corrosion-induced ultrasound signals can be detected with satisfactory signal-to-noise ratio using non-contact AE sensors. Ultrasound waves passing through marine growth showed around 12 dB drop in amplitude when compared to the base plate. A localization algorithm for corrosion induced-ultrasound signals was successfully implemented.

## 1. Introduction

Offshore structures, e.g., wind turbines, photovoltaic, and floating production and storage and offloading units (FPSOs), are key assets for the energy production at sea. These structures are generally designed to withstand environmental loads such as waves, currents, wind, and operational forces. Due to the subsea environment, corrosion has also been regarded as one of main degradation types affecting the integrity of the submerged part of these structures, e.g., monopiles, mooring chains, and pipelines [1,2,3,4]. Additionally, the presence of marine growth on the surface of the submerged structure presents a challenge in detailed assessments of the structural integrity.

Visual inspection can provide a general overview of the structure and possibly allow to determine location and severity of corrosion damage after cleaning of the surface and analysis of high-resolution photographs [5]. However, this technique requires clear water and adequate lightning (possible limiting requirements); it is limited to surface defects and could require further inspection of the damage using more advanced NDT methods (such as Magnetic Particle Inspection (MPI), radiography, or ultrasonic inspection) after surface cleaning [6].

Successful applications of MPI for corrosion inspection and/or monitoring underwater have been reported [5,7,8]. Nonetheless, for MPI, as well as for visual inspection, there is the need of adequate underwater lightning and thorough surface cleaning to allow assessment of the defects [6].

Radiography can readily detect corrosion, and it is typically applied in service to identify and monitor corrosion damage [5]. This technique does not necessarily require prior surface cleaning. However, only small areas can be inspected at a time, no accurate depth information can be derived from the 2D image of the inspected area and the deployment of radiation source can represent a potential health hazard for the operators.

Investigations of ultrasonic technique for detection and imaging of corrosion have been performed [9,10,11]. Ultrasonic testing has proved to be able to locate corrosion damage and estimate its size [5]; nevertheless, the ultrasound source probe typically installed on the material surface (together with a set of receivers) implies surface cleaning, a strong electric energy source, and possible influence of surface roughness on the test results.

The Metal Magnetic Memory (MMM) method [12] has been demonstrated in detecting micro-defects caused by stress concentration (e.g., corrosion pits) in ferromagnetic materials without surface cleaning [13]. The MMM method has been used in recent years to locate corrosion areas and evaluate the corrosion extent [14,15,16]. However, defect identification with the MMM technique is still limited due to various influence factors in testing such as defect shapes, defect depths, material types, etc. [13].

Given the electrochemical nature of the corrosion process, electrochemical techniques find wide applications in the monitoring and controlling of corrosion [17]. Applications of potentiodynamic polarization (PDP) are reported in the literature [18,19,20,21]. However, the PDP is not considered a non-destructive technique and not fit for long-term monitoring [21]. Applications of less destructive (compared to PDP) techniques, such as Linear Polarization Resistance (LPR) and Electrochemical Impedance Spectroscopy (EIS), are reported in the literature as alternatives to the PDP [17,20,21,22,23,24,25,26,27]. Nonetheless, in order to retrieve the corrosion rate at the monitored area, EIS have to be interpreted with the help of Tafel slopes that are usually obtained from the PDP curves [17,21].

The acoustic emissions (AE) technique, which is a passive ultrasound method, is widely recognised as an effective method for monitoring of corrosion damage [28]. Each possible AE source during the corrosion process can be described with its specific properties [28,29,30,31,32,33,34,35,36,37,38,39,40,41,42,43,44]. Hydrogen gas evolution and breakdown of surface oxide films have been pointed out as possible AE sources by Yuyama et al. [35]. The authors also suggested that fracture or decohesion of precipitates, second-phase particles, or non-metallic inclusions should produce detectable AE, as well as microcracking. Recent studies have also focused on discretizing the different corrosion types, e.g., pitting vs. uniform, and characterize the respective possible AE sources [28]. Fregonese et al. [33] observed that, for pitting corrosion, the initiation stage is not significantly emissive, whereas the propagation stage is characterized by high acoustic activity. The authors also suggested that the change of corrosion mode from pitting to uniform corrosion can be followed by the AE technique, as the representative parameters (e.g., rise time and counts number) are drastically affected. In the pitting corrosion process, instantaneous stress changes on the metal surface, rupture of the passivation film, corrosion potential fluctuations, and hydrogen bubble evolution have been suggested as possible mechanisms for AE sources [36,37,38,39,44]. Regarding the uniform corrosion process, hydrogen bubbles release, friction of hydrogen bubbles, and the evolution of corrosion deposits are indicated as predominant sources of AE signals [41]. Investigations on the detectability of ultrasound signals using non-contact AE technique have also been reported in the literature [45,46,47,48].

Inspection and assessment of corrosion damage using the AE technique in submerged steel structure offers high potential. Nevertheless, the so-far developed inspection approaches predominantly share the need to reach the cleaned surface of the assessed structure. Additionally, to the authors’ knowledge, the effect of marine growth on the ultrasound signals has not been sufficiently investigated.

This paper investigates the feasibility of detecting and localising corrosion-induced damage in submerged steel structures using non-contact AE technique without the need to prepare the metal surface, i.e., removing of possible marine growth. Accelerated corrosion experiments have been conducted on two carbon steel plates, one baseline sample, and one covered with artificially made marine growth. A localization algorithm for corrosion-induced ultrasound signals has been implemented to evaluate the feasibility and the accuracy of the proposed method.

The paper has the following structure. The methodology is presented in Section 2. Description of the experiments is given in Section 3. The results are presented and discussed in Section 4, followed by the conclusions in Section 5.

## 2. Methodology

**Figure 1:**
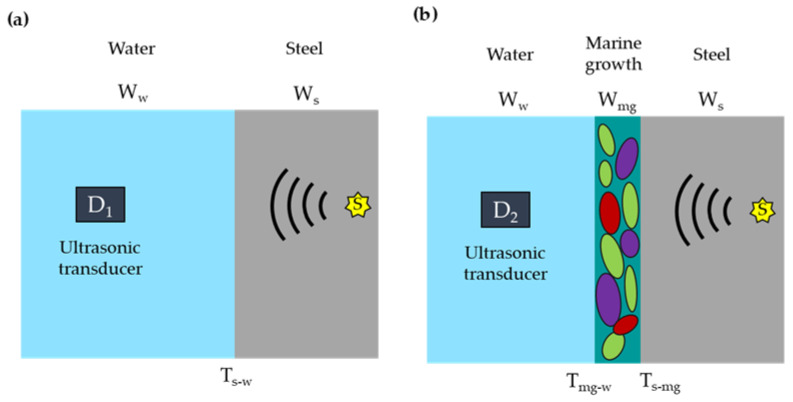
Schematic illustration of primary elastic wave propagating paths (**a**) without marine growth and (**b**) with marine growth.

Figure 1 shows a schematic representation of the wave propagation in two different scenarios: one with a steel structure without marine growth (Figure 1a) and one covered with marine growth (Figure 1b).

In isotropic solids, e.g., steel, ultrasound waves generated from the source, *S*, can propagate as bulk waves (following longitudinal and shear mode) and as guided waves depending on the frequency of the waves and the thickness of the medium [49,50]. When propagating through a homogeneous isotropic fluid, e.g., water, with a constant speed of sound ultrasound waves propagate as pressure waves ruled by the longitudinal wave mode [50,51]. When the ultrasound waves travelling in one medium encounter the boundary of a second medium, reflected and transmitted waves are generated [52]. The ratios of the pressure amplitudes and intensities of the reflected and transmitted waves to those of the incident wave depend on the characteristic acoustic impedance *r* and speed of sound *c* in the two media (and on the incident angle) [53]. Ultrasound wave components with sufficient energy can reach sensor *D*_1_ and *D*_2_. In the frequency domain, the recorded signals *P*_1_ and *P*_2_ can be described as the convolution of the source signal with the transfer functions of the media involved and the interfaces, as in

(1) $$P_{1}=D_{1}W_{w}T_{w-s}W_{s}S+N_{1}$$

(2) $$P_{2}=D_{2}W_{w}T_{mg-w}W_{mg}T_{s-mg}W_{s}S+N_{2}$$

where *W_w_*, *W_mg_* and *W_s_* are the propagation (transfer) functions of water, marine growth and steel. respectively. *T_s-w_* represents the transmission coefficients between steel and water, *T_s-mg_* is the transmission coefficient between steel and marine growth, and *T_mg-w_* the transmission coefficient between marine growth and water. *N*_1_ and *N*_2_ refer to the background noise and the neglected components of the waves. If *N*_1_ and *N*_2_ are neglected, the relative amplitude of the two signals *P*_1_ and *P*_2_ can be described as:

(3) $$P_{2}=\frac{D_{2}}{D_{1}}\frac{T_{mg-w}T_{s-mg}}{T_{w-s}}W_{mg}P_{1}$$

If sensors *D*_1_ and *D*_2_ have the same transfer function, they cancel out, and the resulting quantity becomes an approximation of the attenuation of ultrasound waves passing through marine growth. Assuming normal wave incident, the intensity transmission coefficient is defined as:

(4) $$T=\frac{4r_{2}r_{1}}{{\left(r_{2}+r_{1}\right)}^{2}}$$

where *r*_1_ and *r*_2_ are the characteristic acoustic impedance of two different media, defined as:

(5) $$r=\rho_{0_{i}}c_{i}$$

where $\rho_{0_{i}}$ and $c_{i}$ are the respective characteristics density and speed of sound of the two media.

### 2.1. Corrosion-Induced Acoustic Emissions

Corrosion is typically defined as electrochemical degradation of metals. This structural degradation is mostly driven by the reaction of metal with the surrounding environment. Corrosion mechanism in aqueous solution usually requires three components: a metal (e.g., steel); water; and a corrodent (e.g., carbon dioxide, oxygen, bacteria, hydrogen sulphide, acid, etc.). Corrosion occurs through the operation of coupled electrochemical half-cell reactions, being anodic and cathodic reactions [17]. In the anodic reaction, a given species (e.g., iron) undergoes oxidation with a resulting loss of electrons at the anodic site. While, in the cathodic reaction a given species (e.g., oxygen) undergoes reduction, with the electrons being consumed by the reaction.

Iron dissolution occurs as an anodic reaction, as in:

(6) $$\mathrm{Fe}(s)\underset{\mathrm{Anodic}}{\to }{\mathrm{Fe}}^{2+}{(\mathrm{aq})+2e}^{-}$$

where hydrated iron cations are formed by liberating two electrons.

According to the electroneutrality principle [54], the cathodic reaction will occur simultaneously to consume the electrons released during the anodic reaction in order to keep the electrochemical system in balance.

In neutral or basic water (seawater is usually neutral or weakly alkaline) with a certain amount of oxygen dissolved, the cathodic reaction is as follows [17]:

(7) $$O_{2}{(g)+2H}_{2}{O(l)+4e}^{-}\underset{\mathrm{Cathodic}}{\to }{4\mathrm{OH}}^{-}\left(\mathrm{aq}\right)$$

where dissolved oxygen is reduced to hydroxide ions.

Combining Equation (6) with Equation (7), the corrosion reaction equation in seawater results in:

(8) $${2\mathrm{Fe}(s)+O}_{2}{(g)+2H}_{2}O(l)\underset{}{\to }{2\mathrm{Fe}}^{2+}{(\mathrm{aq})+4\mathrm{OH}}^{-}\left(\mathrm{aq}\right)$$

In the cathode region, iron cations will react with water and/or hydroxide ions to precipitate out of the solution forming iron hydroxides (Equation (9)). After dehydration of the resulting hydroxides, rust (e.g., Fe(OH)_3_, Fe_3_O_4_) is formed. The creation of the latter is described through a series of reactions, as presented in Equations (9)–(11).

(9) $${\mathrm{Fe}}^{2+}{+2\mathrm{OH}}^{-}\underset{}{\to }{\mathrm{Fe}(\mathrm{OH})}_{2}$$

(10) $${4\mathrm{Fe}(\mathrm{OH})}_{2}{+O}_{2}{+H}_{2}O\underset{}{\to }{4\mathrm{Fe}(\mathrm{OH})}_{3}$$

(11) $${4\mathrm{Fe}(\mathrm{OH})}_{3}\underset{}{\to }{\mathrm{Fe}}_{2}O_{3}\cdot H_{2}{O+2H}_{2}O$$

In their pioneering work Yuyama et al. [35] suggested possible AE sources during corrosion process. Hydrogen gas evolution (which can additionally happen due to cathodic reaction in acid solutions) and breakdown of surface oxide films were pointed out as possible AE sources. The authors also suggested that fracture or decohesion of precipitates, second-phase particles, or non-metallic inclusion should produce detectable AE, as well as microcracking.

### 2.2. Localisation of Corrosion-Induced Acoustic Emissions

Different techniques for localizing acoustic emissions sources have been described and compared by Kundu [55]. The approach presented in this research is based on the triangulation technique for isotropic materials with known wave speed of sound reported in the review.

Acoustic emissions source location is estimated based on the difference of signals arrival time at the sensors, given that the location of the sensors is known. On a discretized domain, the time-of-flight *T* between the source on the *i*th grid point and the *k*th sensors is computed as:

(12) $$T_{i,k}=\sqrt{{\left(x_{k}-x_{i}\right)}^{2}+{\left(y_{k}-y_{i}\right)}^{2}+{\left(z_{k}-z_{i}\right)}^{2}}/c_{w}$$

where the subscript *i* refers to the grid number in the mesh, *k* refers to the sensor and *c_w_* is the speed of sound in water (i.e., 1480 m/s at 20 °C [51]).

**Figure 2:**
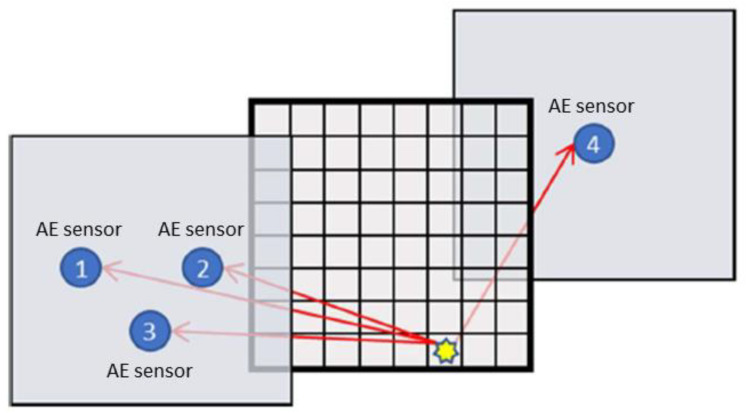
Schematic illustration of localisation approach.

For the specific arrangement of the specimen and the sensors, the problem is simplified to a 2D scheme, where the specimen z-location is known with respect to the sensors. In Figure 2, the specimen is meshed into a number of grid points.

The difference in time of arrival is obtained for the meshed grid points with respect to one of the sensors:

(13) $$\delta T_{i,k,m}=T_{i,k}-T_{i,m}$$

The cross-correlation technique has been used to estimate the time difference of arrival of ultrasound signals at two different sensors. From the recorded signals, differential arrival time is obtained by calculating the time delay between signals recorded by *k*th sensors, and the reference sensor *m*. Using cross-correlation between the recorded signals leads to:

(14) $$\tau_{k,m}=\arg\max(P_{k}*P_{m})(t)$$

Note that alternative methods such as standard threshold crossing technique, wavelet transform, analysis of the LTA/STA (long-time average/short-time average), higher order statistic (HOS) [56], Akaike information criterion (AIC) [57], and Maeda’s equation [58] can also be used to estimate the time of arrival of AE signals [55].

After obtaining both the predicted and the actual differential arrival times, the most probable location of the source signal is obtained by minimizing the error between them:

(15) $$\min(\delta T_{i,k,m}-\tau_{k,m})\;\forall i\in [1,2,\ldots ,N]$$

To improve the reliability of the source localization, a maximum allowable error value is additionally used in Equation (15) [55].

## 3. Experimental Section

Experiments have been conducted to study the possibility of measuring AE signals through marine growth. AE signals amplitude attenuation due to artificially made marine growth has been investigated. The experiments also covered the interaction between marine growth and corrosion induced damage simulated by conducting accelerated corrosion tests. The loss in the material due to corrosion has been assessed using an ultrasound thickness gauge.

### 3.1. Test Specimens

In this study, flat steel plates have been used as test specimens. The plates were made of S235 hot-rolled structural steel, with a carbon content of 0.25% of the total composition.

**Figure 3:**
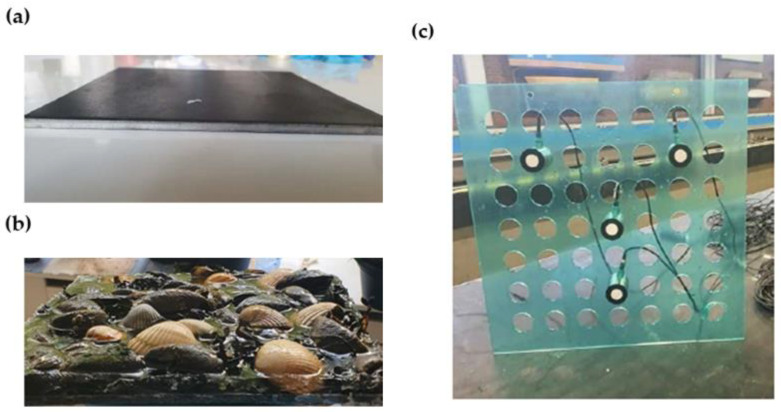
Test specimen (**a**) baseline plate and (**b**) marine growth covered plate. (**c**) Sensor holder with four ultrasound transducers.

Figure 3a,b shows the two carbon steel plates used during the experiments. Dimensions of the plates were 200 × 200 mm^2^, with a thickness of 5 mm. A baseline specimen without marine growth (Figure 3a) has been used to compare the AE signals amplitude against those measured in the case with marine growth covered plate (Figure 3b).

The main drive behind considering artificial marine growth is to facilitate the testing and to obtain estimates for the signal attenuation due to marine growth with hard shells. The composition of this hard marine growth was based on the DNV-GL definition [59]. It contained mussels, shells, bladder-wrack, and seaweed to represent both soft and hard marine growth (Figure 3b). Epoxy resin was used to bind components to the surface of the specimen.

### 3.2. Experiments Setup

In a non-contact inspection arrangement, the sensors have been placed at a fixed distance from the sample. A sensor holder has been designed to keep the sensors at the desired distance and orientation relative to each other and the tested specimen. An acrylic plastic plate (Figure 3c) of 250 × 250 × 4 mm^3^ was used. Laser-cut holes were made to fit the piezoelectric AE transducers. To allow various configuration, 49 (i.e., 7 × 7 grid) holes were cut with a centre-to-centre distance of 50 mm. Figure 3c shows the designed senor holder with four sensors.

**Figure 4:**
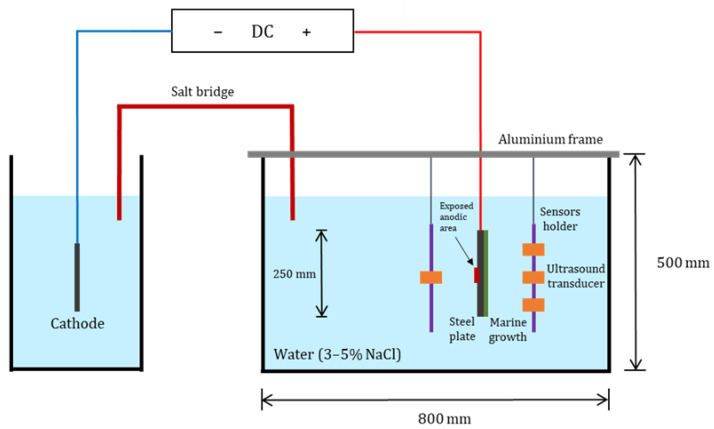
Schematic illustration of accelerated corrosion experiments.

**Figure 5:**
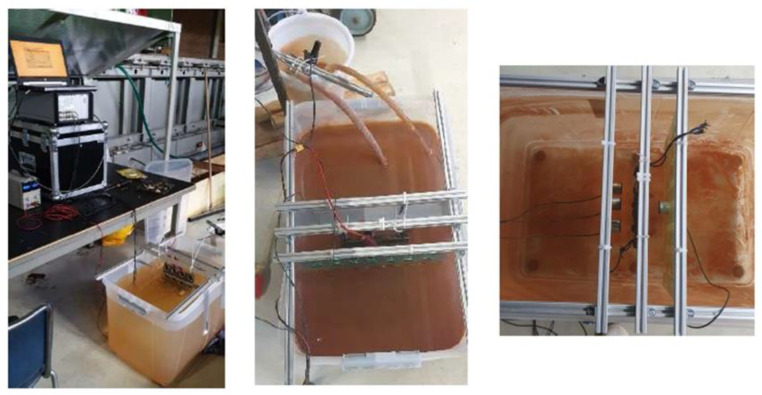
Accelerated corrosion experiments setup.

Figure 4 shows a schematic of the test setup, also depicted in Figure 5. The tests have been executed inside a water container with dimensions of 800 × 500 × 500 mm^3^. An aluminium frame with sliding rails has been used to ensure a fixed distance between the AE sensors and the specimen. Both tested specimens had an exposed anodic area of 2 cm^2^. A direct current has been imposed to accelerate the corrosion process with positive pole connected to the submerged specimen (i.e., anode) and the negative pole connected to a stainless-steel plate (i.e., cathode). Direct current power supply (i.e., 36 V and 5 A) has been deployed. Sodium chloride electrolyte (i.e., NaCl) has been used to ensure sufficient electrical conductivity between the cathode and the anode.

The cathode has been placed in a separate water container to ensure that the acoustic emissions of hydrogen bubbles possibly forming on the stainless-steel plate did not interfere with acoustic emissions generated on the corroding anode. A salt bridge has been made between the two water containers using two PVC tubes. Four piezoelectric AE transducers were used to record the AE signals generated during the corrosion process. Three AE sensors have been placed on one side of the specimen (i.e., marine growth covered side) and the fourth sensor on the other side. The motivation behind this choice was first to achieve better accuracy in the damage localization technique since the waves recorded by all the sensors experienced comparable trajectories and interfaces. Secondly, since corrosion process mainly took place on one side of the plate (i.e., exposed anodic area), it was of interest to compare the detected acoustic emissions on both sides of the specimen. On one side, the acoustic emissions directly propagated through water, whereas on the other side, acoustic emissions propagated through either steel (first specimen) or through steel and marine growth (second specimen).

### 3.3. Accelerated Corrosion on Baseline Specimen

The accelerated corrosion experiment performed on the steel plate without marine growth served as baseline for comparison with the signals measured in the marine growth covered specimen. An electrolyte concentration of 3% NaCl has been used with a direct current of 10 mA and an exposed circular area of 2 cm^2^. Isolation of the rest of the specimen surface was achieved using an aerosol paint with an average thickness of about 10 μm. The duration of the experiment was about three weeks. The specimen has been taken out of the water only once during week one.

### 3.4. Accelerated Corrosion on Marine Growth Covered Specimen

The main objective of the experiment was to measure the corrosion-induced AE in the presence of marine growth. An electrolyte concentration of 5% NaCl has been used. A direct current of 30 mA was imposed on the same exposed area as the baseline plate. Isolation of this specimen was achieved using epoxy paint with an average thickness of about 30 μm. The motivation behind the selection of such covering layer was based on the observation of the first accelerated corrosion experiment, where the aerosol paint could not desirably hold off corrosion leading to the formation of multiple anodic areas. The specimen has been submerged in water throughout the entire duration of the experiment (i.e., three weeks).

### 3.5. Data Acquisition, Management, and Quality Control

**Figure 6:**
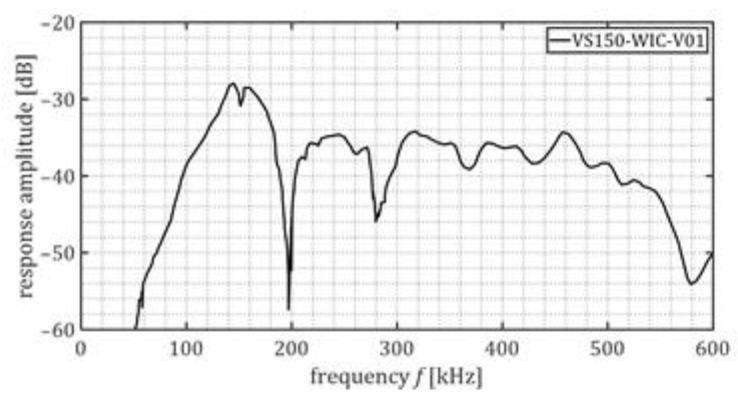
Transfer function of the piezoelectric AE transducers [60].

An AMSY-6 Vallen data acquisition system (with six channels) and four piezoelectric AE transducers, Vallen Systeme VS150-WIC, have been deployed to measure and record the ultrasound waves generated during the corrosion process. The transfer function of the piezoelectric AE transducers is shown in Figure 6.

The sensors were water-tight and able to withstand 60 bar of water pressure. Also, they included an integrated preamplifier with a gain of 34 dB. These AE transducers are characterized by a resonance frequency of 150 kHz (Figure 6). Watertight cables have been used during the experiment.

AE data and AE signals waveforms sampling rate were set equal to 10 MHz during all the reported tests. AE signals waveforms were recorded using a pre-trigger equal to 250 μs during pencil lead breaks and increased to 500 μs during accelerated corrosion experiments (with and without marine growth). No digital filters were applied during the acquisition of AE signals. The AE acquisition threshold was set to 40 dB (relative to 1 μV) during pencil lead breaks and accelerated corrosion experiment of baseplate, and to 35 dB during accelerated corrosion experiment of marine growth covered plate. This choice was motivated by the relatively low amplitude of the corrosion-induced acoustic emissions observed during the experiment performed on the baseplate.

Submerged pencil lead breaks (Hsu-Nielsen source) have been performed as quality control before the accelerated corrosion experiment. The leads have been broken underwater on the surface of the specimen. Both the baseline plate and the marine growth covered plate have been tested. The piezoelectric sensors have been set at a distance where reflections from water container boundaries and water surface would not interfere with the first arrival of the simulated signals. The distance between the specimen and the plane of sensors was 50 mm. Pencil lead breaks have been repeated at a fixed location (in the centre of the plate) on both the specimens.

## 4. Results and Discussion

**Figure 7:**
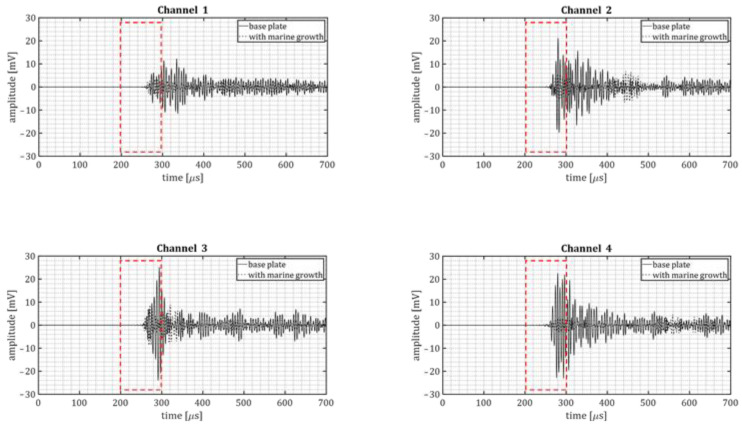
Samples of the processed pencil lead breaks signals for baseline plate and marine growth covered plate. Red dashed box indicates the FFT calculation window, which intends to exclude, as much as possible, the reflections and scatterings of the signals caused by finite dimensions of the tank and specimens.

For quality control, simulated cracks experiments were performed by means of pencil lead breaks. The same sensor layout of corrosion experiments was used for the quality assessment. The signals acquired by the data-acquisition system were stored in an SQL database and later exported to MATLAB. The signals were processed applying a windowing function, and subsequently a frequency-domain bandpass (120–380 kHz) filter to de-noise the signals. Figure 7 shows examples of the processed signals waveforms of pencil lead breaks performed on both plates (baseline and marine growth covered plate).

The drop of the maximum signal amplitude in the time domain differed between the four sensors, as shown in Table 1. The drop of the maximum signal amplitude was defined as:

(16) $$amp_{drop}=10\log_{10}\frac{\max\left|amp_{base}\right|}{\max\left|amp_{mg}\right|}$$

where *amp_base_* and *amp_mg_* refer to the amplitude of recorded signals of the base plate and marine growth covered plate, respectively.

An average *amp_drop_* of about 12.4 dB (considering channels 2–4). The drop of signal amplitude is considered promising and expected to be measurable in realistic conditions. The non-homogenous marine growth layer can explain the difference in amplitude drop. The different dimensions and types of shells may affect the ultrasound wave propagating in different directions. The sensors were placed at different distances and angles from the acoustic emissions source to account for such irregularities.

The variation in the amplitude drop is expected to be mainly due to the details of the positioning of hard shells on each wave propagation path. In practice these variations always exist. Hence, the full range should be considered as possible amplitude drop.

**Figure 8:**
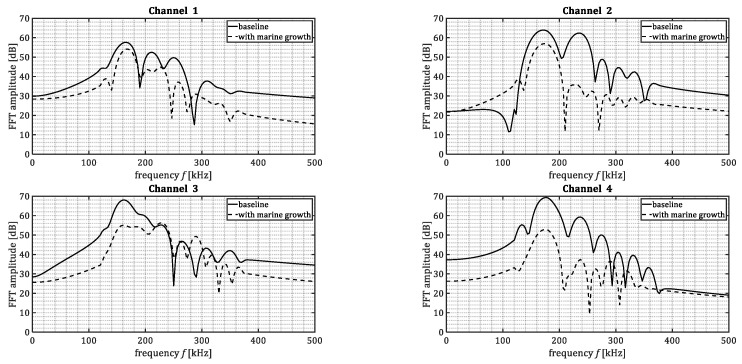
Samples of frequency spectrum of the processed pencil lead breaks signals for baseline plate and marine growth covered plate.

Figure 8 shows the frequency content of the waveforms presented in Figure 7. The time-domain signals were transformed into frequency-domain by means of Fourier transform. The Fourier transform was taken for part of the signal in the time domain, being 85 μs after the first threshold crossing. It was observed that acoustic emissions passing through steel and marine growth layer (channel 2–4) experienced higher attenuation in comparison to the acoustic emissions passing only through steel (channel 1). The level of attenuation was not constant among all the frequencies. The lowest difference was observed mostly in the range of 150–175 kHz. This might be due to the resonance frequency of the used sensors. Additionally, higher frequencies (>175 kHz) show higher attenuation level when passing through marine growth. Reason to that is thought to be the heterogeneous character of the used composition (shells, mussels). It can also be observed that despite the general similarity among the frequency content for the four sensors, there are some irregularities, e.g., frequency content of a pencil lead break signal for the marine growth covered plate. For example, in Figure 8 (channel 3) it is notable that for frequencies above 200 kHz the frequency content is in the same order of the PLB signal of the baseplate. This may be because the frequency content from different PLBs does not match perfectly. Also due to the boundary reflections, additional notches in the frequency domain can be created.

The observation was that ultrasound waves of the simulated signals penetrated through artificial-fabricated marine growth and were successfully detected. A drop in amplitude in comparison with the base plate signals was observed and quantified.

Accelerated corrosion experiments were performed. Each of the experiments took three weeks. The water in the container gradually turned red. After a week, it was quite hard to see through the water. This was due to corrosion deposits in water. For both the baseline plate and the marine growth-covered plate, signal analysis of example recordings in time and frequency domains is presented first. Subsequently, the damage localization results are illustrated.

### 4.1. Corrosion-Induced Acoustic Emissions on Baseplate

**Figure 9:**
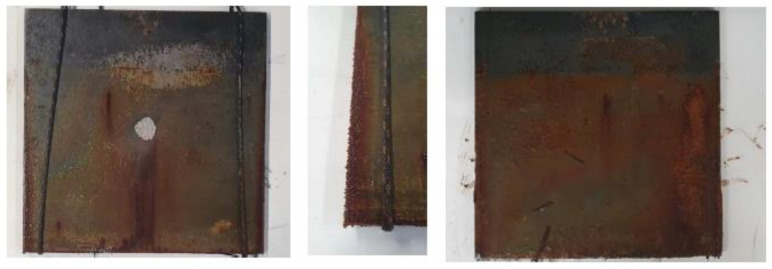
Baseplate after 3 weeks of accelerated corrosion.

Figure 9 shows the corrosion damage for the base plate at the end of the experiment. It can be seen that the main corrosion damage took place at the exposed surface. Other places of corrosion are observed on both sides and at the edges. It appeared that the use of aerosol paint was not sufficient to withstand the corrosive environment it was subject to. Plate thickness loss at mid-plate was measured using an ultrasound thickness measurement probe (Sauter TN 80-0.1 US) and was averaged in the exposed area to be 0.5 mm.

**Figure 10:**
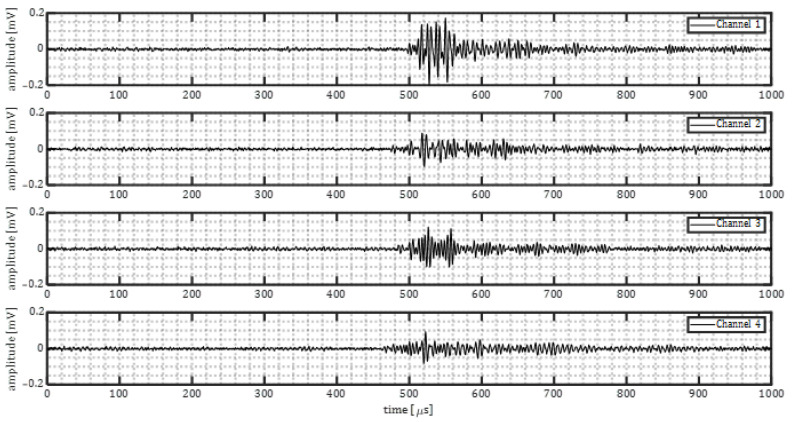
The corrosion induced signals for baseline plate experiment.

Figure 10 shows waveforms from a corrosion event on the baseplate. Channels 2–4 were placed at the fully insulated side of the plate, and channel 1 was placed at the exposed side of the plate. It can be seen that the amplitude of corrosion induced acoustic emission is in the range of 0.08–0.2 mV.

**Figure 11:**
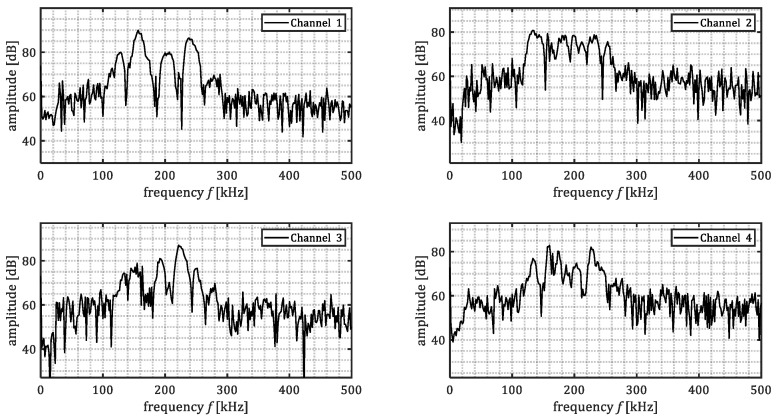
Frequency content of one corrosion event (baseplate).

The frequency content of corrosion-induced acoustic emissions is shown in Figure 11. A general agreement among amplitude peaks is noticed. The peak at 150 kHz is expected because of the resonance frequency of the sensors. Another frequency peak is observed at around 225 kHz for all the sensors. This is expected to be related to the peak frequency excited by the corrosion process.

A damage localization algorithm was implemented to ensure that the analysed signals are corrosion-related. Only signals that occurred inside time-window of 50 μs and came from different sensors were considered as an event. Any event localized with an error higher than 25 μs was regarded as noise. The error was calculated based on the formulation reported in Section 2.

**Figure 12:**
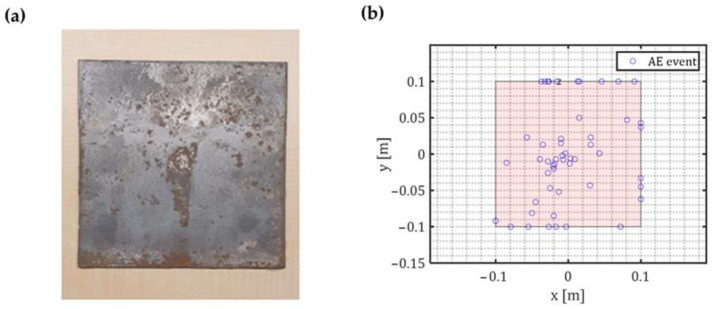
(**a**) Corrosion damage on the baseplate; (**b**) Localisation results on the baseplate.

Figure 12b shows corrosion damage localization results. A total of 52 events were successfully localized. A significant number of events was located around the mid-plate. Although, a number of corrosion events on the edges were also observed (Figure 12a,b).

### 4.2. Corrosion-Induced Acoustic Emissions on Marine Growth Covered Plate

**Figure 13:**
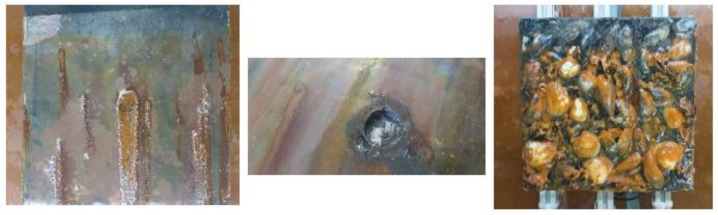
Marine growth covered plate after three weeks of accelerated corrosion.

Figure 13 shows the corrosion damage for the marine growth covered plate at the end of the experiment. It can be seen that the major corrosion damage took place at the exposed surface. Compared to the base plate, better isolation was achieved with epoxy paint and showed more resilience towards the corrosive environment. However, small corrosion spots are noticed due to paint imperfections. Plate thickness loss at mid-plate was measured using ultrasound thickness measurement probe and was averaged in the exposed area to be 3.6 mm. An increase in thickness loss is related to higher anodic current. The increase in current is due to three reasons: smaller anodic area (better isolation), higher applied imposed direct current and electrolyte concentration compared with concentration in the baseline plate experiment (5% NaCl vs. 3% NaCl).

**Figure 14:**
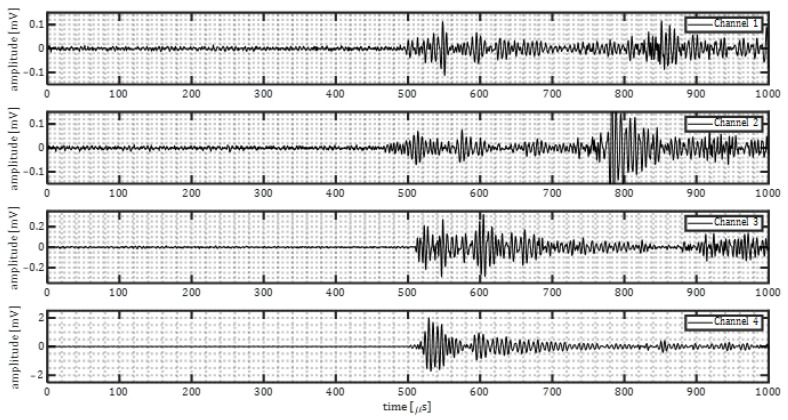
Samples of corrosion-induced signals for marine growth covered plate experiment.

Figure 14 shows waveforms from a corrosion event on the covered plate. Channels 1–3 were placed at one side of the plate (marine growth side), and channel 4 at the exposed side of the plate. It can be seen that the maximum amplitude of corrosion-induced acoustic emission first arrival is in the range of 0.08–0.2 mV.

**Figure 15:**
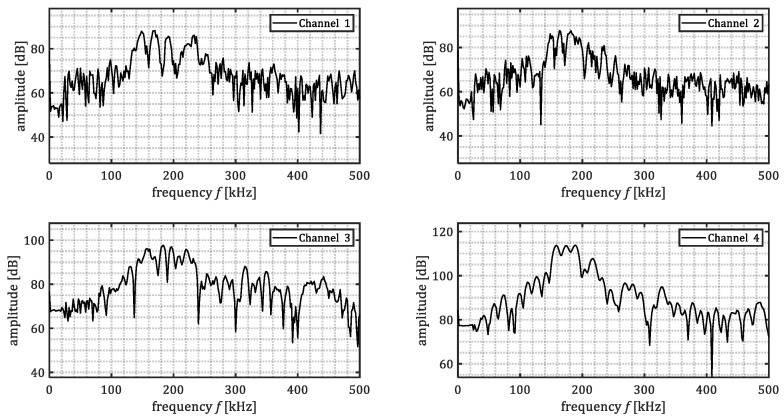
Samples of frequency content of one corrosion event (marine growth covered plate).

Figure 15 shows the frequency contents of the example corrosion event signals in Figure 14. When compared to the frequency content of corrosion-induced signals on the baseplate, the same trend is observed. The peak around 150 kHz is likely due to the senor resonance frequency, and the peak around 225 kHz might be related to the characteristics of corrosion-induced acoustic emissions.

**Figure 16:**
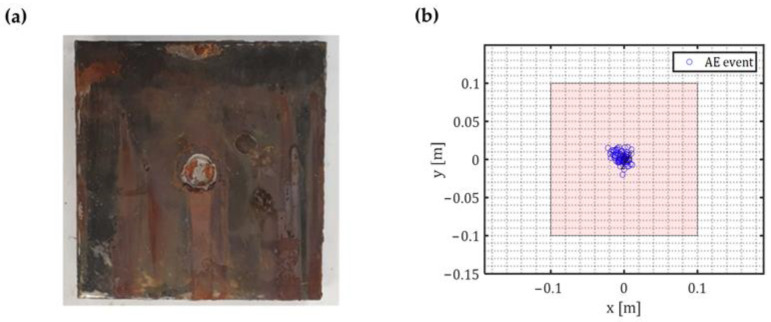
(**a**) Corrosion damage on the marine growth covered plate; (**b**) Localisation results on the marine growth covered plate.

The same damage localization algorithm was implemented as reported in Section 2 with the same criteria used in the previous experiment to ensure accurate event localization. Figure 16b shows localised corrosion damages. A total of 84 events were successfully localized. All the events were located around the centre of the plate. Comparing the results with the actual damage (Figure 16a) a good agreement is observed.

## 5. Conclusions

A specimen with marine growth layer was fabricated by embedding representative natural components and organisms on a steel plate with the help of epoxy as binder. Experiments were conducted to study the possibility of inspection through marine growth. The experiments covered the interaction between marine growth and corrosion induced damage. Corrosion damage was simulated by conducting accelerated corrosion tests.

Results of pencil lead break experiments showed that ultrasound signals can be successfully detected through the fabricated marine growth. Ultrasound waves passing through marine growth showed around 12 dB drop in amplitude in time-domain when compared to the base plate. Also, increased attenuation at higher frequencies was observed. The attenuation caused by marine growth generally depend on its thickness and composition, as they influence the wave propagation properties in the layer and the acoustic impedance mismatch with the adjacent steel material and water.

Results of accelerated corrosion experiments suggest that corrosion-induced ultrasound signals can be detected with satisfactory signal-to-noise ratio using non-contact AE sensors. The recorded corrosion-induced signals had amplitude in the range of 0.08–0.2 mV. A localization algorithm for corrosion induced-ultrasound signals was successfully implemented. The results were verified by comparing localized events with actual damage. The frequency content of corrosion-induced ultrasound waves showed a peak of around 225 kHz for the specimen with and without marine growth.

Although the fabricated marine growth layer is based on a representative composition, the binding material selection may not be fully representative. The chosen epoxy resin is a hard material and does not allow for water ingression into the marine growth layer, which may have an effect on the measured attenuation of the fabricated marine growth. Based on the above-mentioned conclusions and limitations, the following recommendations for the future research are derived. Regarding marine growth characterization, more quantitative attenuation measurements for different types of marine growth is recommended. For future attenuation measurements, it is recommended to use sensors with different frequency ranges to capture a wider frequency spectrum. When artificially modelling marine growth, softer binders may be more representative of real marine growth.

## Figures and Tables

**Table 1:** Maximum amplitudes of acquired signals with an amplitude drop between the baseplate and marine growth covered plate experiment.

	Channel 1	Channel 2	Channel 3	Channel 4
Maximum amplitude (mV) Baseplate	7.7	21.2	25.2	22.6
Maximum amplitude (mV) Marine growth covered plate	3.6	5.6	8.7	3.4
Amplitude drop (%)	53.2	73.6	65.5	85.0
Amplitude drop (dB)	6.6	11.6	9.2	16.5

## Data Availability

The data presented in this study are available upon request from the corresponding author.
